# Advances of super-resolution fluorescence polarization microscopy and its applications in life sciences

**DOI:** 10.1016/j.csbj.2020.06.038

**Published:** 2020-06-26

**Authors:** Long Chen, Xingye Chen, Xusan Yang, Chao He, Miaoyan Wang, Peng Xi, Juntao Gao

**Affiliations:** a Department of Automation, Tsinghua University, 100084 Beijing, China; b MOE Key Laboratory of Bioinformatics; Bioinformatics Division, Center for Synthetic & Systems Biology, BNRist; Center for Synthetic & Systems Biology, Tsinghua University, 100084 Beijing, China; c Department of Biomedical Engineering, College of Engineering, Peking University, Beijing 100871, China; d Department of Engineering Science, University of Oxford, Parks Road, Oxford OX1 3PJ, UK

**Keywords:** Fluorescence polarization microscopy, Dipole orientation, Super-resolution, Biomolecule interactions, Molecular organization

## Abstract

Fluorescence polarization microscopy (FPM) analyzes both intensity and orientation of fluorescence dipole, and reflects the structural specificity of target molecules. It has become an important tool for studying protein organization, orientational order, and structural changes in cells. However, suffering from optical diffraction limit, conventional FPM has low orientation resolution and observation accuracy, as the polarization information is averaged by multiple fluorescent molecules within a diffraction-limited volume. Recently, novel super-resolution FPMs have been developed to break the diffraction barrier. In this review, we will introduce the recent progress to achieve sub-diffraction determination of dipole orientation. Biological applications, based on polarization analysis of fluorescence dipole, are also summarized, with focus on chromophore-target molecule interaction and molecular organization.

## 1 Introduction

Fluorescent molecule has four intrinsic portraits: intensity which reflects the density of fluorescence, wavelength which accounts for the absorption spectrum and emission spectrum, lifetime which characterizes the decaying life of fluorescence, and polarization which reveals the dipole orientation [1], [2], [3], [4]. The polarization property of fluorescence was discovered by French physicist Perrin in 1926 [5], [6]. Under excitation fluorescent molecules absorb and emit photons in the manner of dipole radiation. This phenomenon is called the polarization of chromophore. The fundamental physical principles of fluorescence polarization have been intensively investigated [4], [7], [8].

The orientation of fluorescent molecule is closely related to the targeted subcellular structure. For example, the orientation of protein structure determines the dipole orientation of the fluorophore molecule if the linker between the fluorescent molecule and target protein is rigid enough. Therefore, the spatial direction of the target protein can be reflected by the dipole orientation of fluorescent molecules.

Utilizing the polarization and intensity characteristics of fluorescence molecules, fluorescence polarization microscopy (FPM), also called label-based polarization microscopy, can simultaneously measure dipole intensity and orientation information to study the underlying biological structure organization. For example, FPM is used to monitor the dynamic activation process of membrane proteins [9], to reveal the arrangement of Y-shaped subcomplex unit in nuclear pore complexes (NPC) assembly [10], and to demonstrate the organized and ordered distribution of dipole orientation of actin probes [11], [12], [13]. Other types of cytoskeletons, such as microtubules [13] and septin proteins [14], [15], [16], [17], have also been studied. In addition, FPM with high temporal resolution can be used to observe the molecule dynamics in living cells [18].

Because of the optical diffraction limit, conventional FPM technologies resolve the dipole orientation averaged by multiple fluorescent molecules within diffraction-limited volume. Enabling high-resolution polarization analysis has gradually attracted more and more attention in recent years and some strategies have been developed, such as combining traditional FPM technology with super-resolution microscopy [19], [20], [21] and demodulation [22].

In this paper, the optical setups of FPM based on polarization detection (PD) and polarization excitation (PE) are reviewed. The super-resolution FPMs, including polarization single-molecule localization, polarization demodulation and polarized structured illumination, are introduced thereafter. The applications of super-resolution FPM in life sciences, especially about chromophore-target molecule interaction and molecular organization, are summarized.

## 2 Principles of FPM

The core principle of FPM is to extract dipole moment orientation and higher resolution orientation accesses to more detailed observation. Three issues, containing description of dipole orientation, optical setups of FPM and super-resolution FPM, are exhibited in this section.

### 2.1 Description of dipole orientation

**Fig. 1:**
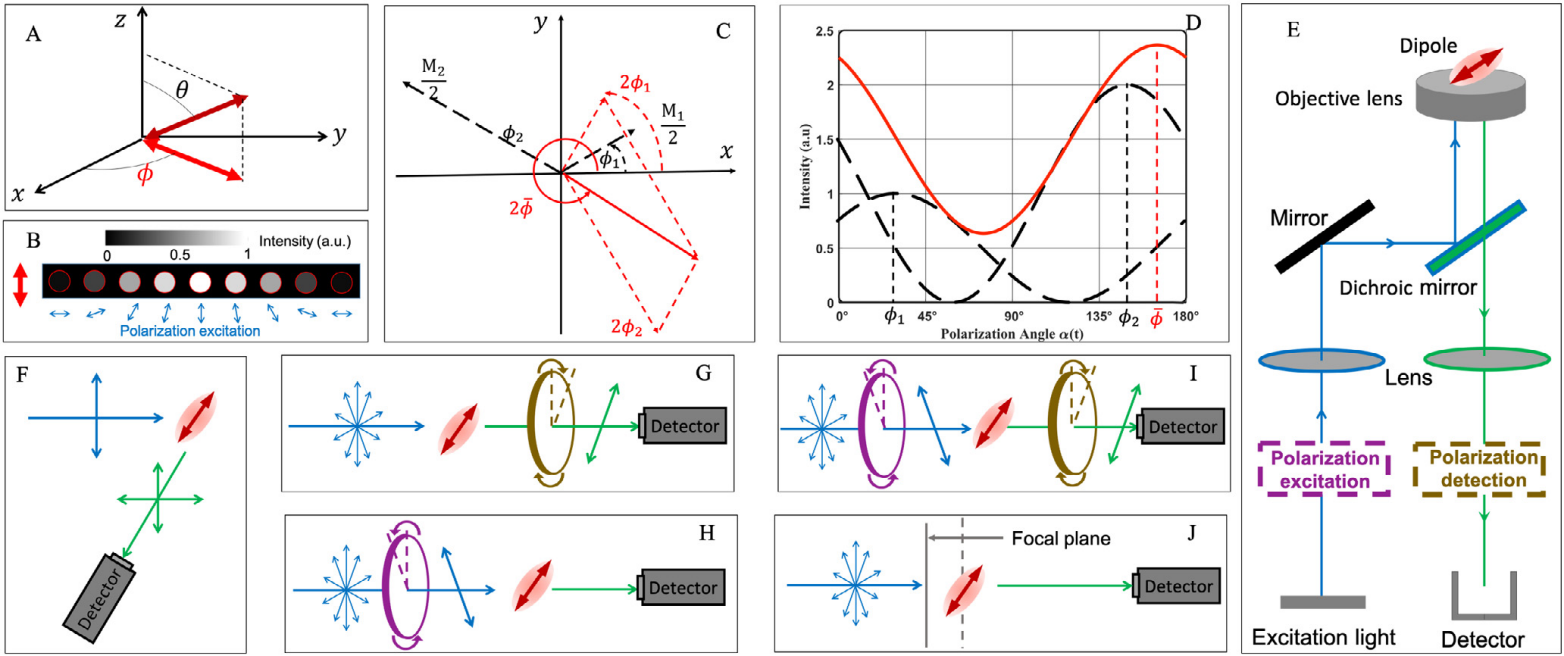
Analytical principle of fluorescent dipole orientation and optical setup. (A) In a spherical coordinate system, an azimuthal angle ϕ and a normal angle θ parameterize the dipole moment orientation. Two red arrows represent 3D and 2D orientation, respectively. (B) The photon intensity is the largest as the dipole orientation is parallel to the direction of the polarized excitation light and the weakest as both directions are perpendicular to each other. (C) The AC components of emission intensity from two dipoles can be added according to the principles of vector addition. Two black arrows and one red arrow refer to two dipoles and their efficient dipole. $\phi_{i}$ and $M_{i}$ represent the orientation and maximum photon of single dipole, respectively. (D) The emission profiles of two dipoles (dashed black curves) and the emission curve of their equivalent ensemble dipole (solid red line) under polarization excitation. (E) Typical optical setup for polarization excitation (PE) and polarization detection (PD) based on wide-field microscope. The modulators of polarization excitation and polarization detection correspond to the purple and brown ovals in Fig. 1G–I. (F) Classical optical scheme of fluorescence anisotropy with one linear polarization excitation, and two directions of observation which are parallel and perpendicular to polarization excitation angle, respectively. (G) Polarization detection setup with isotropic polarization illumination to analyze emission dipole. (H) Polarization excitation scheme to access to absorption dipole. (I) Simultaneous polarization excitation and polarization detection to determine both emission and absorption dipole. (J) Analyzing defocused diffraction pattern to resolve emission dipole. (For interpretation of the references to color in this figure legend, readers are referred to the web version of this article.)

A fluorescent molecule is constituted by atoms, whose spatial arrangement interacts with light and accounts for a defined transition dipole moment. The specific transition dipole moment interacts with target proteins and is an important indicator of potential subcellular organelle organization. The dipole moment orientation is parameterized by azimuth ϕ and normal angle θ in the spherical coordinate system (Fig. 1A). In two-dimensional focal plane, the projection azimuth ϕ indicates the dipole orientation. Under linear polarized excitation, a dipole oscillates a periodic fluorescence signal (Fig. 1B) [23]. Fluorescent molecule has the greatest probability of absorption when its absorption transition dipole moment orientation is parallel to the direction of polarized light.

The absorption probability of the dipole is proportional to cosine-squared (cos^2^) of the angle between the direction of the polarized excitation light and the direction of the absorption dipole. The electromagnetic radiation from a molecular chromophore is proportional to sine-squared (sin^2^) of the intersecting angle between emission dipole orientation and direction of observation. Mostly the absorption dipole moment orientation and emission dipole moment orientation of fluorophores can be regarded as parallel [4], [24], [25].

The emission efficiency of multiple dipoles can be equivalent to that of an effective dipole (Fig. 1C and D). We decompose the emitting intensity of single dipole into the sum of alternating current (AC) component $\frac{M_{i}}{2}cos\left(2\alpha \left(t\right)-2\phi_{i}\right)$ and direct current (DC) component $\frac{M_{i}}{2}$, where $\alpha \left(t\right)$, $\phi_{i}$, and $M_{i}$ represent the polarization angle of excitation light, the orientation and maximum photons of single dipole, respectively. The AC component is regarded as a vector of direction $2\phi_{i}$ with modulus $\frac{M_{i}}{2}$. Therefore, the sum of the AC component by dipole $\phi_{1}$ and $\phi_{2}$ satisfies the vector addition with equivalent dipole direction $\overset{-}{\phi }$, namely,

$$\frac{M_{1}}{2}{cos}^{2}\left(\alpha \left(t\right)-\phi_{1}\right)+\frac{M_{2}}{2}{cos}^{2}\left(\alpha \left(t\right)-\phi_{2}\right)=Acos\left(2\alpha \left(t\right)-2\overset{-}{\phi }\right)+B$$

where,

$$A=\sqrt{{\left(\frac{M_{1}}{2}cos2\phi_{1}+\frac{M_{2}}{2}cos2\phi_{2}\right)}^{2}+{\left(\frac{M_{1}}{2}sin2\phi_{1}+\frac{M_{2}}{2}sin2\phi_{2}\right)}^{2}},$$

$$B=\frac{M_{1}}{2}+\frac{M_{2}}{2},$$

$$\cos2\overset{-}{\phi }=\frac{1}{A}\left(\frac{M_{1}}{2}\cos2\phi_{1}+\frac{M_{2}}{2}\cos2\phi_{2}\right),\sin2\overset{-}{\phi }=\frac{1}{A}\left(\frac{M_{1}}{2}\sin2\phi_{1}+\frac{M_{2}}{2}\sin2\phi_{2}\right).$$

Furthermore, orientation uniformity factor (OUF), defined as OUF = A/B, is purposed to describe the local dipole orientation distribution and wobbling. OUF is represented by the length of stick or double-sided arrows, whose direction corresponds to dipole orientation. OUF is equivalent and proportional to polarization factor (p) *vide infra*.

### 2.2 Fluorescence polarization setups

Both absorption and emission dipole moment orientation are anisotropic. Polarization detection could be used to analyze the emission dipole while polarization excitation to resolve absorption dipole. Combinations of polarization excitation and polarization emission are required to simultaneously analyze both dipole orientations.

The optical setup of wide-field fluorescence microscopy is presented in Fig. 1E and experimental schemes of different categories of FPMs could be implemented in the wide-field geometry (Fig. 1F–J). In addition to wide-field, FPM has also been integrated into various microscopy modalities, such as confocal [26], [27], two-photon [9], [28], [29], [30], [31], total internal reflection fluorescence (TIRF) [32], [33] et al..

In this review, all optical setups of FPM are categorized into four strategies (Fig. 1G-1 J). In the optical path of polarization detection (Fig. 1G), under isotropically polarized illumination fluorescence emission is then separated into different polarization channels. The luminescence polarization modulation can be achieved by Wollaston prism, Thompson prism, and polarization beam splitter [12], [13], [34], [35]. In the optical setup of polarization excitation (Fig. 1H), the polarization modulator adjusts the excitation direction of laser to illuminate the sample, and emission images are detected synchronously by camera. Half wave plates, electro-optical modulators and liquid crystal modulators can accomplish linear polarized light excitation [11], [14], [36]. In Fig. 1I, simultaneous polarization excitation and polarization detection could be used to analyze the transition dipole moments of absorption and emission, as well as the energy transfer between them [7], [25], [37], [38]. In the defocused pattern recognition (DPR) optical path, circularly polarized light is used for the illumination of all dipoles (Fig. 1J). When the sample is several hundred nanometers out of focus from the focal plane of the objective lens, the dipole direction can be extracted from the intensity distribution of fluorescence image [39], [40], [41], [42], [43], [44].

Based on polarization detection scheme, classical FPM has been developed to measure polarization anisotropy [10], [16]. In the optical path, linear polarized light is used to excite in-plane molecules and resultant fluorescence is projected to two orthogonal components whose directions are parallel and perpendicular to the incident light, respectively (Fig. 1F). In fact, classical FPM setup is a special case for simultaneous polarization excitation and polarization detection, with particular linearly polarized illumination and two polarization detection directions.

### 2.3 FPM with super-resolution

Super-resolution microscopy is achieved by three typical methods, stimulated emission depletion (STED) [45], [46], single molecule localization microscopy (SMLM) [47], [48] and structured illumination microscopy (SIM) [18], [49], [50], which bring our vision to a sub-diffraction scale. However, its extension to molecular orientation has not been fully investigated.

Traditional FPM, such as LC-PolScope which uses liquid crystal modulation device to achieve polarization excitation [14], provides polarization information within optical diffraction limited level. To achieve polarization analysis beyond diffraction so that higher resolution brings more detailed and exquisite discoveries, currently there are three types of principles to achieve super-resolution FPM: polarization single-molecule localization, polarized structured illumination and polarization demodulation. The first two types are built upon super-resolution microscopy, namely SMLM and SIM, while the last one is based on sparsity deconvolution. Polarization can be employed in association with STED, by modulating the excitation linear polarization state. However, this inevitably decreases the temporal resolution. One interesting approach is excitation polarization angle narrowing (ExPAN), in which the resolution of super resolution by polarization demodulation (SPoD, referring to details vide infra) can be further improved by enhancing the sparsity through stimulated emission depletion process [22].

Instantaneous FluoPolScope, based on polarization detection setup and single molecule tracking, achieves instantaneous tracking direction and position of fluorophores in live cells [12], [51]. Single molecule localization microscopy is incorporated into FPM to resolve the dipole orientation, such as polar-dSTORM with polarization detection [13] and polar-PALM with polarization excitation [36]. However, super-resolution microscopy based on single molecule localization has minute-level temporal resolution, thus its application in living cells is limited [52]. Because of random activation, off-switching and blinking of fluorescent molecules, absolute intensity differs at different time. When polarization detection setup is incorporated into single molecule localization microscopy, at least two polarization measurements should be conducted simultaneously so that the resolved dipole orientation is reliable.

In conventional SIM setup [49], [53], [54], [55], [56], in order to get high-contrast illumination pattern, the excitation beam must be linearly polarized and its polarization direction should always be parallel to the streak pattern. Due to polarization effect of fluorescence dipoles, the excitation beam is actually structured illumination in both spatial dimension and angular dimension, expanding the observable scope. The dipole model in spatial-angular hyperspace is constructed to simultaneously extract both azimuth and spatial super-resolution information of the fluorescent dipoles [18]. Integrating the advantages of SIM and FPM, pSIM is suitable for rapid imaging of live cells to achieve super-resolution spatio-angular analysis.

Since the fluorescent molecules has polarization dependent response to the excitation beam, Hafi et al. developed SPoD based on polarization excitation setup and sparsity penalty-enhanced estimation by demodulation (SPEED) algorithm [22]. Integrating deconvolution was proposed to resolve comparable result with SPoD by integrating the modulation sequence from SPoD and applying sparsity regularization [57]. Therefore, whether polarization modulation can yield additional resolution information is interrogated. Zhanghao et al. combined advantages of SPoD and FPM to measure both intensity and orientation beyond diffraction limit, namely super-resolution dipole orientation mapping (SDOM) [11], [58]. With both deconvolution and orientation mapping steps in SDOM, the polarization modulation information could be fully utilized to get super-resolution images of intensity and dipole orientation, which reveals that polarization mainly contributes to additional resolution information by detecting dipole orientation and addresses the dispute. SDOM-derivative methods were developed thereafter. For example, SERS-SDOM achieved surface-enhanced Raman scattering (SERS) super-resolution imaging of gold nanoparticles as a technology extension of SDOM [59], and group-sparsity-based SDOM (GS-SDOM) realizes three-dimensional co-localization analysis about the overlapping of cross fiber bundles as an algorithm improvement [60].

A comparison of different super-resolution FPMs is carried out in Table 1, with respect to FPM optical scheme, strategy to achieve super-resolution, number of polarization modulation, spatial and temporal resolution. The advantages and limitations of different techniques are compared according to whether they could access to complex sample with dense labelling, single molecule detection and living imaging. For example, Instantaneous FluoPolScope is only applied to image simple sample with sparse labelling. Polar-dSTORM/PALM could not be employed in live samples. SDOM and pSIM are able to achieve super-resolution living cell imaging of complex samples while they are limited to detect single molecule orientation due to ensemble labeling.

**Table 1:** Comparison of different super-resolution FPMs.

Technique	FPM optical scheme	Strategy to achieve super-resolution FPM	Number of polarization modulation	Spatial resolution	Temporal resolution	Advantages	Limitations
Instantaneous FluoPolScope	PD	Polarization single molecule	4	~200 nm	0.1 s	Single molecule, living cell	Simple sample
Polar-dSTORM	PD	Polarization single molecule	2	~20 nm	Several minutes	Single molecule, complex sample	Fixed sample
Polar-PALM	PE	Polarization single molecule	4	~20 nm	Several minutes	Single molecule, complex sample	Fixed sample
SDOM and its derivatives	PE	Polarization demodulation	10	50 ~ 100 nm	Second	living, complex sample	Ensemble molecules
pSIM	PE	Polarized structured illumination	3	~100 nm	Sub-second	living, complex sample	Ensemble molecules
Polarization detection (PD) and polarization excitation (PE) are categorized by the implementation of polarization modulation in the optical setups. The typical setup of the former one is to utilize polarization analyzer to image the sample fluorescence polarization, and the typical setup of the latter one is to use rotational linear polarized beam to excite the sample.							

## 3 Biological applications of FPM

FPM detects both fluorescence and orientation information, which is essential for a multitude of biological observations and further understanding of cellular functions and processes. Super-resolution FPM is developed recently to bring the conventional diffraction limited FPM to a whole new scale, so that the ensemble dipole number can be reduced, and the orientation can be detected at a higher precision. Two categories of biological applications are demonstrated here: chromophore-target molecule interaction, and molecular organization.

### 3.1 Chromophore-target molecule interaction

In life sciences, fluorescent protein and chemical dye are the most commonly used chromophores. FPM imaging can reveal the polarization interaction between the chromophore and target molecule.

**Fig. 2:**
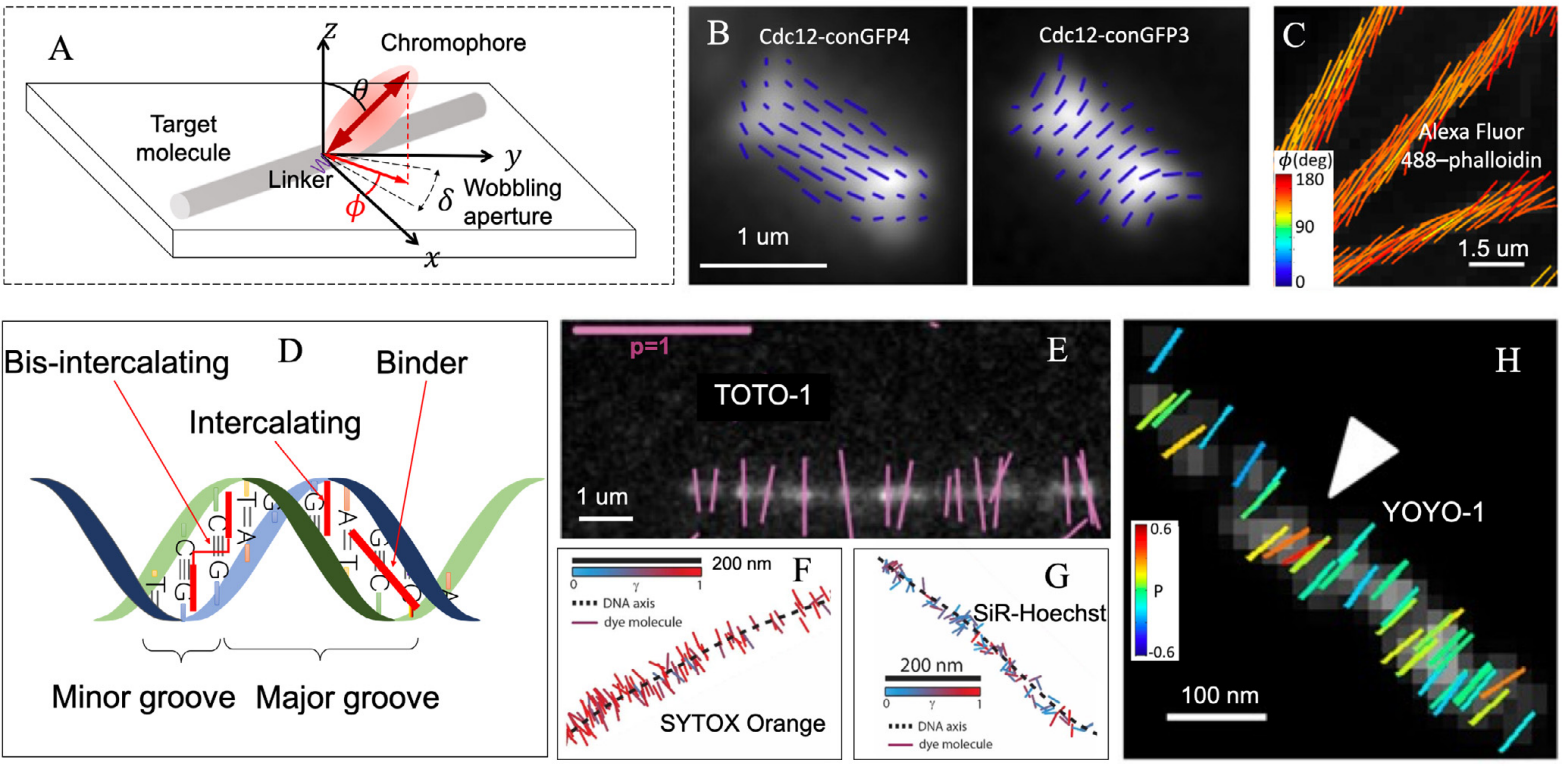
Characterizing of chromophore-target molecule. (A) Diagram of chromophore (pink oval) linking to target molecule (shallow grey rod). (B) The orientation imaging of hourglass structure with Cdc12-conGFP3 and Cdc12-conGFP4 constructs fused into septin, which exhibits orthogonal orientation relationship of the two constructs (image adapted from Ref. [14]). (C) The polar-dSTORM imaging of Alexa Fluor 488 labelled actin fiber (image adapted from Ref. [13]). The angular aperture ϕ is pseudocolored. (D) Schematic diagram of the binding mode of the bis-intercalating, intercalating dyes and groove binding dyes. (E) Fluorescent polarization image of lambda phage DNA stained with TOTO-1 (image adapted from Ref. [12]). (F) DNA imaging probed by intercalating dye SYTOX Orange. (G) *In vitro* interaction between DNA and groove-bound dye SiR-Hoechst. Fig. 2F and G are adapted from Ref. [36]. (H) Local bending (marked by arrowhead) of the DNA strand, probed by bis-intercalating YOYO-1, is detected by polarization analysis (image adapted from Ref. [13]). Orientation of single dipole/pixel is represented by direction of a stick in Fig. 2B, C and Fig. 2E–H. In Fig. 2E and H, polarization factor (p) is defined as $p=\frac{I_{\|}-I_{⊥}}{I_{\|}+I_{⊥}}$, where $I_{\|}$ and $I_{⊥}$ refer to two detected orthogonally polarized intensities. Polarization factor relies on tilting and wobbling of fluorophore and p∈[0,1], where 0 and 1 represent isotropic and fully anisotropic, respectively (referring to OUF *vide supra*). (For interpretation of the references to color in this figure legend, readers are referred to the web version of this article.)

To create a rigid linker between green fluorescence protein (GFP) and septin, one part of the α-helix from N terminus of truncated GFP can be fused to another part of the α-helix from C terminus truncated CDC3, to form a complete α-helix with enough rigidity for PFM imaging. In the FPM imaging of budding yeast (*S. cerevisiae*), dipole orientation of Cdc12-conGFP4 construct orients perpendicular to septin filament while Cdc12-conGFP3 orients parallel to the filament (Fig. 2B). Perpendicular polarization of Cdc12-conGFP4 and Cdc12-conGFP4 is revealed by FPM, which supports the rotation of 90°along the helix fiber every amino acid in the α-helix [15].

On the other hand, chemical dyes were investigated by super-resolution FPM intensively. The phalloidin–dye links of different flexibilities while labelling actin stress fibers in fixed cells are investigated [11], [13]. Alexa Fluor 488, Alexa Fluor 568 and Atto 565 conjugate with actin protein parallelly to the filament direction while Atto 633 labels perpendicularly to it. Alexa Fluor 647 labelled in actin looks completely isotropic, indicating that the dye is not applicable to image actin filament organization. Fig. 2C shows the imaging of Alexa Fluor 488 labelled actin fibers with parallel labeling between dye and fiber direction. The structure of double-stranded DNA can be effectively detected and different types of dye-DNA interactions can be characterized. Bis-intercalating dyes, such as TOTO-1 and YOYO-1, are firmly aligned perpendicular to the DNA axis (Fig. 2E and H) [12], [13], [61]. SYTOX Orange is one type of intercalating dye that can be inserted between adjacent base pairs and labeled approximately perpendicular to the DNA axis (Fig. 2F) [18], [36], [62]. Groove-bound dye SiR-Hoechst exhibits heterogeneous molecular sequences along DNA fiber (Fig. 2G) [36].

### 3.2 Molecular organization

FPM imaging could be used to study the structure behavior and dynamics of the target protein. Dipole orientation measurement of single molecule gives biologists an unparalleled insight into polymeric systems. In this review, we focus on three types of molecular organizations: macromolecule aggregation, biological filament order (cytoskeleton and DNA fiber) and single particle dynamics, where FPM can provide a better insight view.

**Fig. 3:**
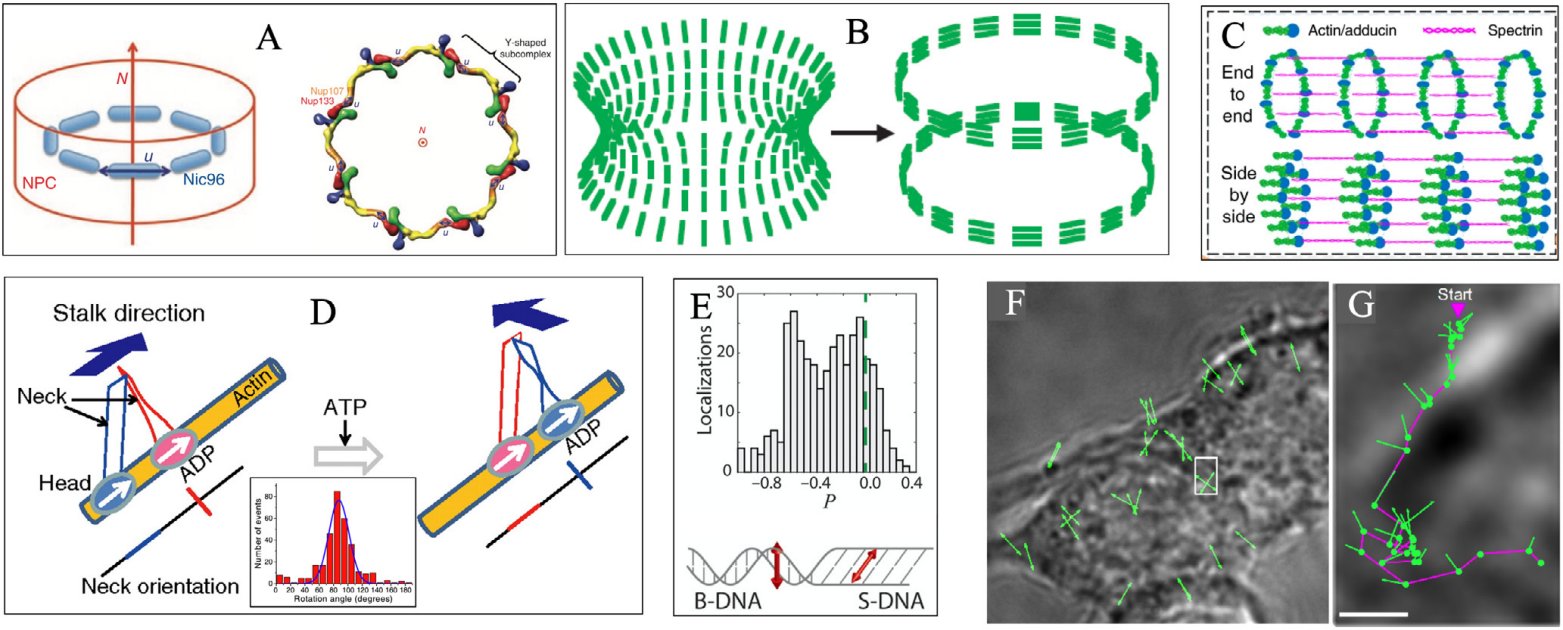
Structural organization observed with FPM. (A) Both long axes (marked by u) of yeast Nic96 and human Nup133–Nup107 orientate approximately perpendicular to the nucleocytoplasmic axis (marked by N), which supports flat head-to-tail ring arrangement formed by the Y-shaped subcomplexes (image adapted from Ref. [10]). (B) Structural dynamics of septin during yeast division from hourglass structure to double ring structure (image adapted from Ref. [16]). (C) In the imaging of the phalloidin-labeled actin ring in neuron axons, pSIM reveals the side-by-side organization of short actin filaments to form actin ring structure (image adapted from Ref. [18]). (D) Myosin motor rotates ~ 90° every step while walking on actin filament (image adapted from Ref. [64]). Two ovals and two ribbons express Myosin V and indicate the head and neck, respectively. The orientations of the two domains are marked with red line and blue line, respectively. Inset is the histogram of the average rotation with peak at 87° (n = 320). (E) Perpendicular and tilted intercalators occur at the transition and are flanked by B-DNA and S-DNA, respectively (image adapted from Ref. [65]). (F) Polarization imaging of SERS particles in living macrophages is presented and the direction of the arrow represents the azimuth of SERS particles. (G) Motion trajectory and rotation information of single SERS particle. Scale bar: 1um. Fig. 3F and 3G are adapted from Ref. [59]. (For interpretation of the references to color in this figure legend, readers are referred to the web version of this article.)

Via fluorescence anisotropy analysis, FPM unveils a relatively parallel arrangement of human nucleoporin Nup133–Nup107 and yeast nucleoporin Nic96 to the nuclear envelope plane. This observation supports the “head-to-tail ring” model that Y-shaped subcomplexes form an octameric ring through interaction between the end and the short arms of subcomplexes (Fig. 3A) [10].

Nanoscale structural imaging of fundamental cytoskeleton and DNA fiber is exhibited by super-resolution FPM and more detailed structures could be detected. During budding, yeast septin proteins are arranged in an orderly manner to form short filaments and rotate 90° along the plane of the membrane, from hourglass structure to double ring structure (Fig. 3B) [11], [16]. The long axis orientation of the actin filament subunits is demonstrated as parallel to the axon shaft, overturning the “end-to-end” structural assumption of adducin-capped actin filaments (Fig. 3C) [18], [63]. Polarization analysis of single dipole reveals the rotational walking of myosin along actin filament [33], [41], [64]. Ohmachi et al. find that myosin V rotates ~90° around its own axis for each movement step via FPM based on polarization detection setup along four polarization angles (Fig. 3D) [64]. Super-resolution spatio-angular imaging displays the accurate dynamics of GFP labelled microtubules in U2OS cell [18]. Cruz et al. observed a local double-stranded DNA bundle, where the direction of YOYO-1 does not follow the average direction of the bundle, indicating the local bending in the DNA strand (Fig. 2H) [13]. With dipole orientation mapped into super-resolution, FPM and DNA manipulation are combined to investigate structural basis of the DNA conformation changes. Independent observations of both intercalators (SYTOX Orange) and bis-intercalators (YOYO-1) provide the same experimental evidence that base pairs are inclined within mechanically stretched DNA. This demonstrates that, compared with the standard conformation of Watson-Crick B-DNA, S-DNA has substantially tilted conformation of base pairs (Fig. 3E) [65].

Wang et al. synthesized SERS nanoparticles with sensitive polarization effect and without photobleaching. SERS-SDOM effectively realizes nanoscale particle tracking to provide trajectory and rotational changes with nanometer spatial resolution in living cells (Fig. 3F and G) [59]. During the retrograde flow in living cell, instantaneous FPM imaging tracks both position and orientation of AF488-phalloidin. Sharp changes in the cytoplasmic structure of AF488-phalloidin along F-actin is observed, revealing the heterogeneous distribution of actin fibers and demonstrating the dynamic molecular interactions which underpin macromolecular assembly mechanism [12].

## 4 Conclusion and outlook

FPM excavates the polarization property of fluorescent probe to supplement orientation information for the study of target protein structure. However, suffering from optical diffraction, conventional FPM is difficult to resolve orientation with high resolution. Recently super-resolution FPMs have been achieved to zap nanoscale orientation information, which is essential for a more detailed study of the polarity, orientational order of intracellular proteins, and thus understanding of life processes. In this review, we focus on the novel advances of technology and application in super-resolution FPM. There are three strategies to accomplish super-resolution FPM, including polarization single molecule localization, polarization demodulation and polarized structured illumination. Biological applications, with the special focus on chromophore-target molecule interaction and molecular organization, are summarized herein.

From perspective of signal processing, polarization modulation can contribute to information enhancement with two more components – phase and period, in addition to the common amplitude which corresponds to intensity. Dipole orientation refers to the phase of polarization modulation and structural information at nanoscale could be accessed by pushing FPM into super-resolution, which brings a new dimension for super-resolution imaging and greatly enriches the texture details of fluorescence microscopic imaging. If over two periods of polarization data are acquired, there is great potential to improve signal-to-noise ratio (SNR) of polarization modulation images with optical lock-in detection to separate the periodical signal from optical intensity with different frequencies, such as auto-fluorescence, stochastic noise, sampling noise and so on [22], [66]. The improvement of SNR would contribute to image analysis, including orientation extraction.

Accurate determination of fluorescence dipole orientation/anisotropy is vitally important for development of FPM. However, the anisotropy would be affected by the energy transfer between fluorescent molecule and its environment. To discern the contribution of energy transfer to measured anisotropy is an important issue. Control measurements, such as red-edge excitation etc, could be applied to isolate the effect of fluorescence resonance energy transfer (FRET) from total anisotropy [7], [67], [68]. 2D dipole orientation is mainly focused in this review and at least three polarization acquisitions are needed for 2D single dipole orientation measurement. As shown in Fig. 1b of Ref. [18], there are three independent spectral components in Fourier domain, where the ±1st frequency component contains the dipole orientation information. Since most sensors are not polarization sensitive, each detective image is the linear combination of these three components, and the coefficient is related to the direction of the excitation beam. When the dipole excited by linear polarized light, it is equal to be sampled once in dipole orientation axis. Therefore, to fully separate these three components and to resolve the dipole orientation, at least three polarization extraction are required.

Due to the lack of axial information, the out-of-focus dipole will cause significant averaging error and affect the 2D measurement. Therefore, 3D dipole orientation determination is very necessary. Several aforementioned FPM techniques can measure 3D dipole orientation. For defocused pattern analysis, the PSF library of 3D orientations can be calculated. Then the 3D orientation of the dipole can be fitted with a matching algorithm [39], [41]. For polarization detection methods, with four polarization channels detected, the 3D dipole orientation can be determined [69]. Both of these two methods are single-molecule based so the temporal resolution is limited. Resolving 3D dipole orientation with high spatiotemporal resolution remains challenging and to be addressed.

Novel biological applications are expected to boost the development of FPM. On the other hand, with more and more advanced super-resolution FPM developed, wider and more elegant biological applications could be investigated. For example, super-resolution FPM with high temporal resolution, fast imaging speed and being highly compatible with live cell imaging could access more elaborate structural dynamics study. However, much more progress is expected. For example, the detailed septin organization during budding yeast, between hourglass and double ring structure, is still not observed and interpreted yet. In immunofluorescence microscopy, since there are multiple linkers between fluorescent molecule and secondary antibody, primary antibody and secondary antibody, anti-body and the target to label the target protein, the distribution of dipole orientations is usually in random order and is not able to represent structure information. To introduce FPM into immunostaining, it is vitally important to further develop more molecular probes that bind target with strong polarization fluorescence.

Incorporating multiple modalities into FPM is promising. Built upon confocal, FPM is used in studying conformation change by measuring fluorescence anisotropy [26], [27], [70]. Fluorescence correlation spectroscopy (FCS), also based on confocal, is applied in measuring viscosity, orientation and local chemical environment [71], [72]. Taking advantage of both analysis of fluorescence anisotropy and correlation spectroscopy has the potential to better investigate molecular conformational changes at single molecule level in living cell. The optical aberrations would affect the precision of the extracted vector information of the dipole. Thus, adaptive optics [73], [74] – the powerful technique for aberration correction  – may play an important role in the further extension usage of FPM, such as in 3D dipole information extraction or deep tissue vector imaging. Furthermore, label-free polarization microscopy – such as widely-used Mueller matrix microscopy [75], [76], [77], [78] – is also a powerful tool for detecting/differentiating microscopic structures, therefore it can be used to monitor physiological changes of tissue samples [79], [80], [81], [82]. There of course exist scopes for FPM to have a future comparison in certain obtained information with that acquired via label-free polarization microscopy – which might be complementary and mutual authentication. Besides, correlative light and electron microscopy combine the advantages of two microscopy modalities to study high-resolution specific events in the cellular environment, which is an important research field of current microscopic imaging [83], [84]. FPM provides new structural insight in biological imaging, which could be combined with electron microscopy to generate novel effective tools for biologists.

## Declaration of Competing Interest

The authors declare that they have no competing interest.
